# Comparison of linear and undulating periodization resistance training on athletic capacities and health promotion: a systematic review and meta-analysis

**DOI:** 10.3389/fpubh.2026.1707627

**Published:** 2026-03-05

**Authors:** ZhiYu Zhang, Xudong Ya, Xinyi Zhao, Ziyao Liu, Jiaxin Luo, Yujia Liu, Yifeng Bu

**Affiliations:** 1 Institute of Physical Education, Jiangsu Normal University, Xuzhou, Jiangsu, China; 2 School of Vocational and Continuing Education, Ningxia Normal University, Guyuan, Ningxia, China

**Keywords:** athletic capacity, blood glucose, blood lipid, body composition, linear periodization, undulating periodization

## Abstract

**Objective:**

This study aims to compare the effects of linear periodization (LP) and undulating periodization (UP) resistance training on athletic ability, body composition, blood lipid, and blood glucose.

**Methods:**

We searched PubMed, Embase, Scopus, and Web of Science to collect relevant studies comparing the health-promoting effects of LP and UP up to July 20th, 2025. Two authors independently extracted and coded the data. The TESTEX Scale was employed to assess the quality of included studies. Egger’s test and sensitivity analysis were conducted to evaluate the robustness of results. Subsequent subgroup analyses were carried out based on participants’ age, sex, training duration, and obesity status.

**Results:**

A total of 29 studies met the inclusion criteria. The results showed that LP and UP had comparable effects on enhancing athletic capacity, improving body composition, and regulating blood glucose and insulin resistance. Further subgroup analysis indicated that UP was superior to LP in increasing lean body mass among obese individuals and achieving short-term weight loss goals. In contrast, LP was more appropriate for long-term weight loss. The sole study involving only males suggested that UP was more effective than LP in reducing insulin resistance in men.

**Conclusion:**

LP and UP demonstrated similar effects on enhancing athletic capacity, improving body composition, and regulating blood glucose and insulin resistance. UP was superior to LP in improving body composition among obese individuals and attaining short-term weight loss goals. Training load regiment can be prescribed according to specific objectives in practical applications.

## 1 Introduction

World Health Organization 2020 guidelines recommended at least 150 min of moderate-intensity aerobic physical activity or 75 min of vigorous-intensity aerobic physical activity weekly, or an equivalent combination thereof, but also moderate or greater intensity involve all major muscle groups resistance training (RT) (1). The healthy promotion induced by RT was widely reported, including but not limited to, bone and muscle development, improved cardiovascular health, reduced blood pressure and low-density lipoprotein cholesterol (LDL) (2). However, the physiological adaptations to RT vary considerably depending on the specific training protocol employed, particularly with respect to load manipulation strategies (3).

Periodization is a popular structure approach that emphasizes the variation of training intensity and volume to improve athletic performance and achieve health promotion (4). The most common periodization models are the linear periodization model (LP) and undulating periodization model (UP). LP usually initiates with high training volumes and low intensities and gradually progresses toward low training volumes and high intensities (5, 6), while UP arranges training loads by increasing and decreasing intensity and volume, with the alterations occurring within the same week; that is, the variation of training components is more frequent and lasts for shorter periods, and can be categorized by adjusted frequency into daily undulating periodization (DUP) and weekly undulating periodization (WUP) (7, 8).

Previous studies and meta-analyses comparing the two loading regiments have chiefly examined maximal strength and hypertrophy, yet the findings remain inconsistent. Harris et al. (3) found no difference between LP and UP in improving upper- or lower-body strength. Conversely, Caldas et al. (9) and Moesgaard et al. (10) reported that UP was superior for increasing maximal strength, though not for power and muscle endurance. These studies generally were limited by small samples and short durations, with subgroup analyses stratified by potential moderating variables notably absent. Whether larger sample sizes and longer training duration studies included and further subgroup analyses would alter the conclusions remains unknown, necessitating an updated synthesis. The physiological effects of RT extend considerably beyond muscular strength augmentation; RT significantly increases muscle mass and upregulates glucose transporter type 4 (GLUT4) expression, thereby enhancing skeletal muscle insulin sensitivity (11). Furthermore, the accretion of lean tissue amplifies the regulatory capacity of skeletal muscle in glucose and lipid metabolism, establishing a positive feedback loop that reinforces metabolic homeostasis. However, relatively few studies have examined the effects of distinct training load regimens on broader health indicators, including body composition, blood lipid profiles, and glucose metabolism, with extant findings demonstrating considerable heterogeneity. Foschini et al. (12) showed that while both training periodization approaches positively impacted body weight, body mass index (BMI), and muscle endurance, only the UP reduced insulin concentration and homeostatic model assessment for insulin R (HOMA-IR). In contrast, Ahmadizad et al. (13) demonstrated that both LP and UP effectively reduced HOMA-IR, body fat percentage, and waist circumference. Consequently, additional research is required to clarify the broader health effects of these two loading regiments. Therefore, this study aimed to conduct a meta-analysis to summarize and analyze the existing literature comparing LP and UP training. The objectives were to elucidate the differences in the impacts of various training load regimens on different aspects of athletic capacities and health promotion, and to further investigate whether distinct training load regimens are applicable to different populations and diverse training purposes. The main findings of this study could provide practical strategies for formulating reasonable load allocation plans for periodized RT for different training goals and demographic groups.

## 2 Methods

The review was conducted in accordance with the PRISMA (Preferred Reporting Items for Systematic Reviews and Meta-Analyses) guidelines.

### 2.1 Search strategy

Studies published before March 20th, 2025 were located using searches of PubMed, Embase, Scopus and Web of Science databases. Combinations of the following search terms were used: “linear,” “undulating,” “non-linear,” “non linear,” “periodized,” “periodization,” “periodization training,” “periodized exercise program,” “periodized physical exercise,” “randomized controlled trial,” “strength,” “muscle strength,” “power,” “muscular strength,” “maximal strength,” “1RM,” “muscle hypertrophy,” “muscle mass,” “non-periodized,” “block periodization,” “obesity,” “body weight,” “overweight,” “BMI,” “body fat rate,” “atherosclerosis,” “figure dimension,” “insulin resistance,” “cholesterol,” “physical fitness,” “athletic ability,” and an updated search was performed on July 20th, 2025. Duplicate publications were removed, and reference lists from retrieved articles were manually reviewed for additional publications not discovered based on database searches.

### 2.2 Eligibility criteria

The inclusion criteria were as follows: (1) peer-reviewed publications; (2) comparison between LP and UP protocols under distinct RT periodization models, with documented training frequency, intensity, and load configurations for each group; (3) reporting of pre- and post-intervention means with standard deviations (SD) or standard errors of the mean (SEM); (4) human participants; (5) intervention duration exceeding 2 weeks; (6) publication in English.

The study was excluded for the following reasons: (1) absence of a direct comparison between LP and UP; (2) single-group design without a comparative arm; (3) insufficiently detailed training protocol; (4) unavailability of data required for effect size (ES) computation.

### 2.3 Study selection and data extraction

Two reviewers extracted the data independently using a standardized Excel template. Disagreements were resolved by a third party. Data extracted were the author’s last name, year of publication, participants’ characteristics including age, which categorized as adolescent (<18 years) or adult (>18 years), sex, exercise prescriptions (duration, intensity, frequency, type, and duration). LP was defined as a linear increase in training intensity and volume per week throughout the intervention, when there was a nonlinear weekly increase, it was classified as WUP, and daily nonlinear increases were categorized as DUP. Outcomes including athletic capacity, body composition, physiological and biochemical markers (blood glucose, blood lipids, and blood pressure). If relevant information was not provided in any given study, the corresponding author was contacted and asked for the data by email.

### 2.4 Bias assessment

“Tool for assessment of study quality and reporting in exercise” (TESTEX) scale was used to assess the quality of studies by two reviewers independently. The scale consists of five possible points for study quality and 10 for study reporting (14). Higher scores mean better study quality and reporting. Disagreements in scoring between reviewers were resolved by a third party.

### 2.5 Statistical analysis

Analyses were carried out using StataMP 18 to measure the ES and 95% confidence intervals (CI). Mean difference (MD) was calculated when the same measurement criterion was used; otherwise, standardized mean difference (SMD) was calculated, ES of 0.2, 0.5, and 0.8 were considered to represent small, moderate, and large differences when using SMD, respectively. Publication bias was evaluated through visual inspection of funnel plot asymmetry and quantitatively assessed using Egger’s test. Given that only 2 studies assessed sprint speed, funnel plot analysis was deemed inappropriate for this outcome; consequently, sensitivity analysis alone was employed. Heterogeneity for the between-study association was evaluated using the *I*^2^ statistic. In terms of statistical significance for *I*^2^, values of 25 to ≤50% were considered low heterogeneity, 50 to ≤75% moderate heterogeneity, and >75% high heterogeneity. Testing for an overall effect (Z score) was regarded as significant at *p* < 0.05. Sensitivity analysis was conducted using a leave-one-out approach to assess the robustness of the effect size. Subgroup analyses were also performed to explore variables that might have influenced training effectiveness, based on participants’ age, sex, training duration, obesity status, and training experience. Regarding the classification of training experience, the absence of standardized criteria in the literature necessitated adherence to the operational definitions employed in the original studies. Participants categorized as “untrained” were predominantly defined as those with no prior engagement in systematic resistance training programs.

## 3 Results

### 3.1 Study selection

**Figure 1:**
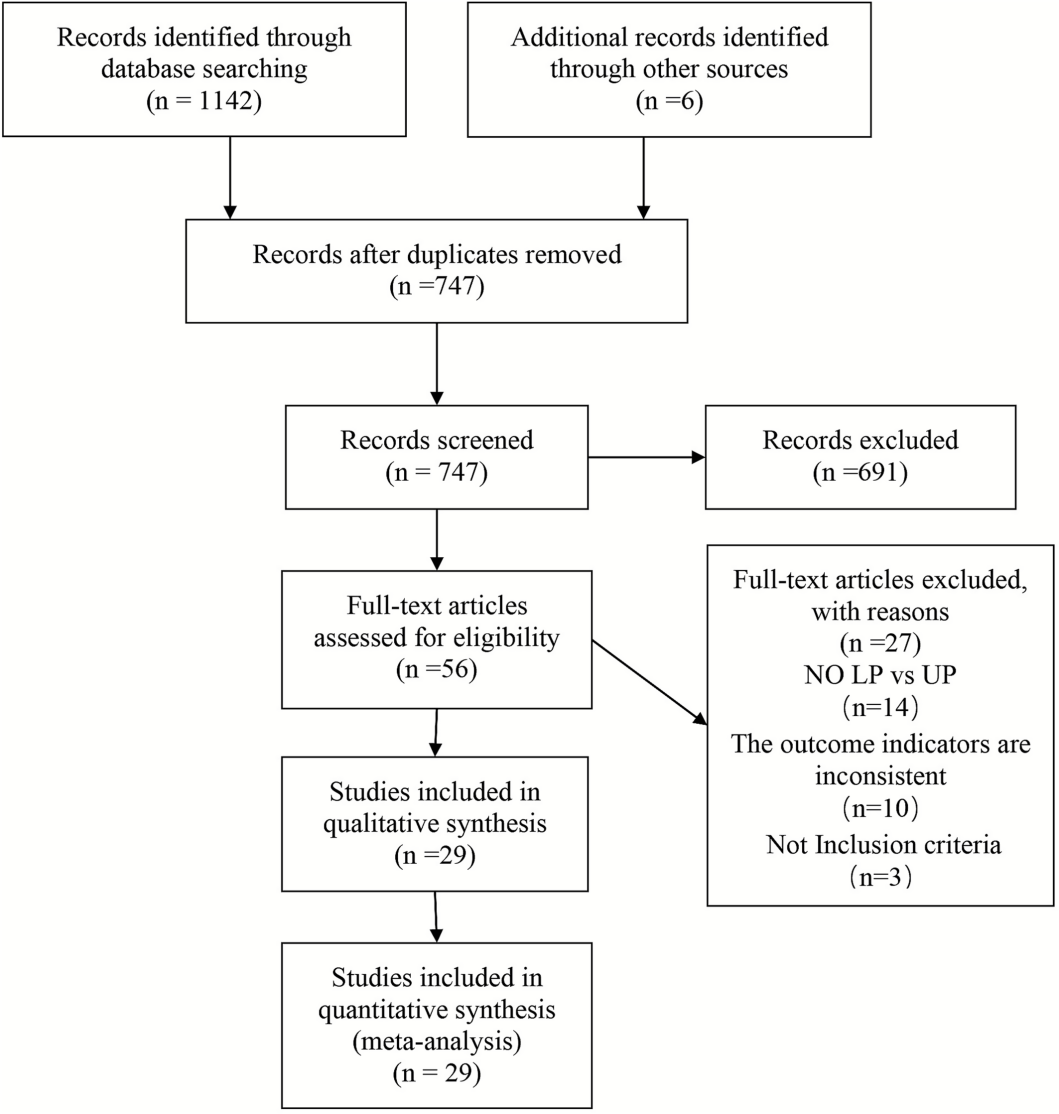
PRISMA flow diagram of the study selection process.

The literature search strategy is outlined in Figure 1. In our initial database search, we identified 1,142 potentially eligible articles, with a further six identified through manual searches. Following the removal of duplicate studies, 747 studies were obtained by preliminary screening based on the titles and abstracts, and 691 articles were excluded, based on the inclusion criteria. The remaining 56 papers underwent full-text screening, at which point 27 further studies were excluded. The final analysis included a total of 29 studies.

### 3.2 Characteristics of the included studies

The included studies involved a total of 704 participants of both sexes. Of these, 17 studies included male participants exclusively, while 5 were limited to female participants. Participants’ mean ages spanned from 14 to 80 years. All studies incorporated RT. One study included both DUP and WUP, and two studies only conducted WUP, and the rest were compared between DUP and LP. Training duration varied between 6 and 28 weeks, and training frequency ranged from two to five sessions per week. Individual training sessions lasted between 33.8 and 90 min. Among the 29 studies, 23 assessed upper limb pushing ability, and 25 evaluated lower limb squat strength. Two studies assessed sprint speed, and 7 reported explosive power. Regarding body composition, body weight, BMI, body fat percentage, and fat-free body weight were examined in 9, 4, 8, and 7 studies, respectively. Blood glucose was evaluated in 3 studies, and insulin resistance parameters in two studies. The effects of different training load regimens on triglycerides, low-density lipoprotein, and blood pressure were reported in one study, respectively. Details of the included studies are presented in Table 1.

**Table 1:** Characteristics of included studies.

Study	Country	Group	Age	Gender (M/F)	Inclusion criteria	Intensity	Frequency	Duration	Outcomes
Baker et al. (1994) (38)	Australia	LP	20.2 ± 1.2	8/0	Male weightlifting instructors with at least six months of weightlifting training experience. Achieve 1RM bench press and 1RM squat greater than their body weight.	Week 1–4: 10RM; Week 5–8: 8RM; Week 9–12: 6RM	3	12	Fat-free body weight↑: LP = UP; VJ↑: LP = UP; 1RM squat↑: LP = UP; 1RM bench press↑: LP = UP
		UP	21.4 ± 5	5/0		Alternate between 10, 8, 6, and 3RM every two weeks.	3	12	
Rhea et al. (2002) (6)	America	LP	21 ± 2.3	10/0	All subjects reported participating in a strength-training program (at least 2 days per week) for a minimum of 2 years LP before beginning the study.	Week 1–4: 8RM; Week 5–8: 6RM; Week 9–12: 4RM	3	12	Bench press strength↑: LP = UP; squat strength↑: LP = UP
		UP		10/0		Day 1: 8RM; Day 2: 6RM; Day 3: 4RM	3	12	
Hoffman et al. (2003) (39)	America	LP	18 ~ 20	28/0	All subjects had between 1 and 3 years of RT experience.	Day 1: 80%1RM; Day 2: 80%1RM	2	12	1RM bench press↔: LP = UP; 1RM squat↑: LP > UP
		UP				Day 1: 70%1RM; Day 2: 90%1RM	2	12	
Rhea et al. (2003) (40)	America	LP	21 ± 2.4	10/10	1–5 years training experience before beginning the study	Weeks 1–5: 25RM; Weeks 6–10: 20RM; Weeks 11–15: 15RM	2	15	1RM squat↑: LP = UP
		UP	21 ± 1.9	10/10		Change the intensity every exercise day, 20RM; 15RM; 10RM; Repeat continuously for 15 weeks	2	15	
Buford et al. (2007) (41)	America	LP	22.29 ± 3.98	5/4	Subjects reported prior weight training experience, but detrained for 2 months.	Weeks 1–3: 8RM; Weeks 4–6: 6RM Weeks 7–9: 4RM	3	9	Body fat percentage↓: LP = DUP/WUP; Bust circumference↔: LP = DUP/WUP; Leg circumference: LP = DUP/WUP; bench press strength↑: LP = DUP/WUP; squat strength↑: LP = DUP/WUP
		WUP		6/3		Weeks 1/4/7: 8RM; Weeks 2/5/8: 6RM Weeks 3/6/9: 4RM	3	9	
		DUP		7/3		Day 1: 8RM; Day 3: 6RM Day 5: 4RM	3	9	
Peterson et al. (2008) (42)	America	LP	21.9 ± 1.8	7/0	Fire fighters in good physical condition.	Weeks 1–3: Hypertrophy and endurance Weeks 4–6: Basic and functional strength; Weeks 7–9: RFD and PP development	3	9	1RM bench press↑: LP = UP; 1RM squat↑: LP = UP VJ↑: LP = UP; SBJ↑: LP = UP 40-yard sprint↓: LP = UP; BM↑: LP = UP Bust circumference↑: LP = UP; Biceps dimension↑: LP = UP; Thigh circumference↓: LP = UP
		UP		7/0		Day 1: Upper body endurance/hypertrophy; lower body strength; Day 2: Upper body strength; lower body power/speed; Day 3: Upper body power/speed; lower body endurance/hypertrophy. Following the overload principle.	3	9	
Hartmann et al. (2009) (43)	Germany	LP	23.98 ± 3.14	13/0	Subjects reported strength training experience in the bench press with a minimum 1RM of100 kg.	Week 1–10: Muscle hypertrophy phase, 5 × 8–12RM; Week 11–14: Strength-power phase, 5 × 3–5RM	3	14	1RM bench press↑: LP = UP; MVC bench press↑: LP = UP
		UP		14/0		Monday: Power - 5 × 3–5RM; Wednesday: Hypertrophy - 5 × 8–12RM; Friday: Endurance - 5 × 20-25RM	3	14	
Hoffman et a. (2009) (44)	America	LP	NM	34/0	Experienced resistance trained American football players of an NCAA Division III football team	Week 1-4: hypertrophy phase (9–12RM) week5-11: strength phase (6–8RM) week12-15: power phase (1–5RM)	4	15	BM↓: LP > UP; 1RM squat ↑: LP = UP 1RM bench press↑: LP = UP; VJ↑: LP = UP
		UP				Strength Phase: 3–5RM strength training and 1–2RM Olympic lifts; Hypertrophy Phase: 9–12RM strength training and 5–6RM Olympic lifts.	4	15	
Kok et al. (2009) (45)	Australia	LP	20 ± 2	0/10	Not trained for 6 months.	Weeks 1–3: 10RM; Weeks 4–6: 6RM Weeks 7–9: 40% of 1RM	3	9	1RM squat↑: LP = UP 1RM bench press↑: LP = UP
		UP		0/10		Day 1: 10RM; Day 2: 6RM Day 3: 30–40% of 1RM	3	9	
Monteiro et al. (2009) (16)	Brazil	LP	27.6 ± 2.7	9/0	Healthy males trained at least 4 days/week in the past 2 years.	Mid-Cycle 1: 12–15RM; Mid-Cycle 2: 8–10RM; Mid-Cycle 3: 4-5RM; Mid-Cycle 4: 12–8-4RM	4	12	BW↔: LP = UP Body fat percentage↔: LP = UP Fat-free body weight↔: LP = UP 1RM squat↑: LP = UP 1RM bench press↑: LP = UP
		UP	28.1 ± 2.9	9/0		First cycle: 12–15RM (first 2 sessions), 8–10RM (last 2 sessions); Second cycle: 4-5RM (first 2 sessions), 12–15RM (last 2 sessions); Third cycle: 8–10RM (first 2 sessions), 4-5RM (last 2 sessions); Fourth cycle: 12–8-4RM	4	12	
Prestes et al. (2009) (7)	Brazil	LP	21.5 ± 8.3	20/0	Strength trained at least 4 times/week using 3 sets of 8–10 RM for 12 months.	Weeks 1–4: 12RM, 10RM, 8RM, 6RM; Weeks 5–8: Same as Weeks 1–4 Weeks 9–12: Same as Weeks 1–4	4	12	bench press strength↑: LP = UP squat strength↑: LP = UP
		UP		20/0		Odd Weeks (1, 3, 5, 7, 9, 11): Days 1–2: 12RM; Days 3–4:10RM; Even Weeks (2, 4, 6, 8, 10, 12): Days 1–2:8RM; Days 3–4:6RM	4	12	
Vanni et al. (2009) (46)	Brazil	LP	39.6 ± 0.41	0/14	35–44 years old Caucasian premenopausal women. Not trained in the previous 6 months.	Weeks 1–4: 20-18RM; Weeks 5–8: 18-16RM; Weeks 9–12: 16-14RM Weeks 13–16: 14-12RM; Weeks 17–20: 12-10RM; Weeks 21–24: 10-8RM Weeks 25–28: 8-6RM	3	28	1RM squat↑: LP = UP 1RM bench press↑: LP = UP
		UP		0/13		Weeks 1–4: 20-18RM; Weeks 5–8: 12-10RM; Weeks 9–12: 8-6RM Weeks 13–16: 12-10RM; Weeks 17–20: 8-6RM; Weeks 21–24: 12-10RM Weeks 25–28: 8-6RM	3	28	
Apel et al. (2011) (47)	Canada	LP	22 ± 2.3	14/0	All subjects had previous weightlifting experience (6–11 months) using free weight and machine resistance before.	Weeks 1–2: 57% 1RM (Adaptation); Weeks 3–7: 62, 73, 76, 79% 1RM Week 8: Rest/Recovery; Weeks 9–11: 75, 78, 80% 1RM; Week 12: Rest/Recovery	Weeks 1–2: 3 times/week Weeks 3+: 4 times/week	12	Squat strength↑: LP = UP; bench press strength↑: LP = UP; Leg stretch↑: LP = UP High pull-down↑: LP = UP; DB Shoulder press↑: LP = UP
		UP		14/0		Weeks 1–2: 57% 1RM; Weeks 3–11: 62, 79, 73, 76, 80, 75, 78% of 1RM; Week 12: Recovery	First 2 weeks: 3x weekly Afterwards: 4x weekly	12	
Miranda et al. (2011) (48)	Brazil	LP	26 ± 6	10/0	Men received entertainment training voluntarily	Weeks 1–4: 8–10RM; Weeks 5–8: 6–8RM; Weeks 9–12:4–6RM	4	12	1RM squat↑: LP = UP 1RM bench press↑: LP = UP
		UP	26.5 ± 5	10/0		Day 1: 8–10RM; Day 2: 6–8RM Day 3: 4–6RM	4	12	
Lima et al. (2012) (49)	Brazil	LP	28 ~ 35	0/10	Non-obese, not trained 6 months before the initiation of the study	Weeks 1–12: 30RM-25RM-20RM-15RM	Training week: 4 times/week Recovery week: 2 times/week	12	Body fat percentage↓: LP = UP; Fat mass↓: LP = UP; Fat-free body weight↑: LP = UP
		UP		0/10		Weeks 1,3,5,7,9: Day1 - Day2: 30RM Day3 - Day4:25RM; Weeks 2,4,6,8,10,12; Day1 - Day2:20RM Day3 - Day4:15RM		12	
Simão et al. (2012) (50)	Brazil	LP	29.8 ± 1.9	10/0	Not performed trained for at least 6 months before the start of the study	Weeks 1–4: 12–15RM; Weeks 5–8: 8–10RM; Weeks 9–12: 3–5RM	2	12	1RM bench press↑: LP = UP
		UP	30.2 ± 1.1	11/0		Weeks 1–2: 12–15RM; Weeks 3–4: 8–10RM; Weeks 5–6: 3–5RM Weeks 7–12: Day 1: 12–15RM Day 2: 8–10RM; Day 3: 3–5RM	2	12	
Ahmadizad et al. (2013) (13)	Iran	LP	23.4 ± 0.6	8/0	Healthy overweight men, and not trained for at least 12 months prior to the study.	Weeks 1–2 (adaptation weeks): intensity at 50–60% of 1RM; Weeks 3–8: intensity at 50,60,65,70,75 and 85% of 1RM, respectively.	3	8	BW↔: LP = UP; body fat percentage↓: LP = UP BMI↔: LP = UP; Fasting blood glucose↓: LP = UP Fasting insulin index↓: LP = UP; 1RM bench press↔: LP = UP; 1RM squat↔: LP = UP
		UP		8/0		Weeks 1–2: Adaptation weeks with 50–60% of 1RM; Weeks 3–8: Day 1: 55% of 1RM; Day 2: 70% of 1RM; Day 3: 85% of 1RM	3	8	
Franchini et al. (2015) (51)	Brazil	LP	18–35	6/0	18–35 years old brown or black belt judo athlete trained at least 3 times per week more than 6 months	Weeks 1–2: 3–5RM; Weeks 3–5: 80% of 1RM; Weeks 6–8: 15-20RM	5	8	BW↔: LP = UP Standing long jump↔: LP = UP 1RM squat↑: LP = UP 1RM bench press↑: LP = UP
		UP		7/0		Mon & Tue: 3–5RM; Wed & Thu: 80% 1RM; Fri: 15-20RM	5	8	
Harries et al. (2015) (3)	Australia	LP	14 ~ 18	8/0	Sub-elite Youth Rugby athlete	Weekly intensity gradually increases from 75 to 90% of 1RM.	2	12	BW↔: LP = UP; Skeletal muscle mass↔: LP = UP Body fat percentage↔: LP = UP; 1RM squat↑: LP = UP; 1RM bench press↑: LP = UP
		UP		8/0		Weekly strength training follows a trend of increasing intensity, with two sessions per week at different intensity levels, the second session being more intense than the first.	2	12	
Prestes et al. (2015) (52)	Brazil	LP	60~	0/20	Female, sedentary, BMI ≤ 30.0 kg/m^2^, age ≥60 years old.	Weeks 1–4: 12-14RM; Weeks 5–8: 10-12RM; Weeks 9–12: 8–10RM Weeks 13–16: 6–8RM	2	16	BW↓: LP>UP; BMI↔: LP = UP; Neck circumference↔: LP > UP; Waist circumference↓: LP > UP; Hip circumference↓: LP > UP; Body fat percentage↔: LP = UP; Lean body mass↔: LP = UP; Body fat content↔: LP = UP; Fat-free body weight↔: LP = UP; Irisin↔: LP = UP; Tool-like receptor 4↔: LP = UP; Brain-derived neurotrophic factor↓: LP<UP; Interleukin cells 15↓: LP = UP; bench press strength↑: LP > UP; squat strength↑: LP = UP; Chair stand↑: LP = UP; Arm curl↑: LP = UP; TUG↓: LP = UP; 6-MWT↔: LP = UP; Flexibility↑: LP = UP
		UP		0/19		Over 16 weeks, strength training intensity alternates between 12-14RM, 10-12RM, 8–10RM, and 6–8RM each week.	2	16	
Ullrich et al. (2017) (53)	Germany	LP	24.3 ± 2.6	6/5	Soccer, handball, basketball, tennis, field hockey athletes at top regional division level, have performed in their sports during the previous 5 years, and were experienced with total body strength training and plyometric exercises for 5.1 ± 2.2 years.	Weeks 1–2: Body weight Weeks 3–4: 15% increase Weeks 5–6: 30% increase	3	6	BM↔: LP = UP; BMI↔: LP = UP; Leg circumference↔: LP = UP
		UP		6/5		Three weekly sessions alternating between body weight, 15% body weight, and 30% body weight.	3	6	
Buskard et al. (2018) (54)	America	LP	58 ~ 80	10/6	Not trained in the past six months	Weeks 1–4: Build strength at 80% of 1RM.; Weeks 5–6: Recovery phase with functional recovery exercises.; Weeks 7–10: Develop power.; Weeks 11–12: Recovery phase with functional recovery exercises.	3	12	Bench press strength↑: LP = UP; squat strength↑: LP = UP; bench press power↑: LP = UP squat power↑: LP = UP; GJST↓: LP = UP; LT↓: LP = UP; FTST↓: LP = UP; 5 s CS↓: LP = UP; TUG↓: LP = UP; 6 MWT↓: LP = UP
		UP		4/10		12-week plan: Day 1 - strength building at 80% 1RM; Day 2 - power development; Day 3 - functional recovery.	3	12	
Souza et al. (2018) (55)	America	LP	25 ± 7	9/0	Engaged in sports but not undergoing regular strength and endurance training for at least 6 months	Week 1–4: 12RM; Week 5–8: 8RM Week 9–12: 4RM	2	12	1RM squat↑: LP = UP
		UP	24.4 ± 5.2	8/0		Weeks 1–4: 12RM and 6RM; Weeks 5–8: 10RM and 6RM; Weeks 9–12: 8RM and 4RM	2	12	
Mahmoud et al. (2021) (56)	Iran	LP	28 ~ 43	0/10	Non-menopausal, no regular exercise training for the past 6 months	Weeks 1–4: 15RM; Weeks 5–8: 10RM Weeks 9–12: 3–5RM	3	12	BW↓: LP = UP; BMI↓: LP = UP; Body fat percentage↓: LP = UP; Muscle mass↑: LP = UP Glucose↓: LP = UP; Insulin index↓: LP = UP Insulin resistance↓: LP = UP; Lymphocyte↑: LP = UP Neutrophil↓: LP = UP; IGF-1↑: LP = UP Interleukin-7↑: LP = UP; 1RM squat↑: LP = UP 1RM bench press↑: LP = UP
		UP		0/9		Phase 1: Weeks 1–2: 15RM Weeks 3–4:10RM; Weeks 5–6: 3–5RM Phase 2 (Weeks 7–12): Day 1: 3–5RM Day 2:10RM; Day 3:15RM	3	12	
Rosell et al. (2021) (57)	Spain	LP	24.3 ± 4.2	16/0	Having a RT experience ranging from 1 to 3 years (with at least 1–3 sessions per week)	Average speed slows weekly in the same relative intensity.	2	8	CMJ↑: LP = UP 1RM squat↑: LP = UP
		UP	22.4 ± 2.7	16/0		Alternate weekly speed for same relative intensity.	2	8	
Borges-Silva et al. (2022) (58)	Spain	LP	22.5 ± 2.8	21/0	Not participated in any strength training for at least three months	Performed at 70% of 1RM. Load can be progressively increased based on 1RM.	2	8	Body fat content↓: LP<UP; Lean body mass↑: LP > UP; Lean body mass of the upper limbs↑: LP = UP1RM bench press↑: LP = UP; 1RM Supine deadlift↑: LP = UP
		UP		20/0		Strength at 90, 70, and 50% of 1RM alternates to develop max strength and hypertrophy.	2	8	
Gonçalves et al. (2024) (59)	Brazil	LP	21.57 ± 2.02	10/0	Participants had 1.46 years–6.46 years’ experience with calisthenic type RT as part of military physical training (MPT).	Weeks 1–3: 12–15RM; Weeks 4–6: 8–10RM; Weeks 7–9: 3–5RM	2	9	Lean body mass of the lower extremities↑: LP = UP squat 1RM↑: LP<UP
		UP		11/0		In the 9 weeks, the intensity of each training session every week alternates among 12 - 15RM, 8 - 10RM and 3 - 5RM.	2	9	
Riscart-López et al. (2024) (60)	Spain	LP	23.7 ± 3.2	12/0	Strength-trained men had RT experience ranging from 1.5 to 4 years and were apt to perform the SQ exercise.	Week 1: 50% 1RM; Week 2: 55% 1RM Week 3: 60% 1RM; Week 4: 65% 1RM Week 5: 70% 1RM; Week 6: 75% 1RM Week 7: 80% 1RM; Week 8: 85% 1RM	2	8	1RM squat↑: LP = UP CMJ↑: LP = UP 20 m sprint time↔: LP = UP
		UP	22 ± 4	10/0		Weeks 1–2: 50 and 60%; Weeks 3–4: 55 and 70%; Weeks 5–6: 65 and 80% Weeks 7–8: 75 and 85%	2	8	
Vargas-Molina et al. (2024) (15)	Spain	LP	64 ± 2.1	10/8	Older adults people with no training experience at all	Perform 3 sets of 8–10 reps each.	3	8	Fasting blood glucose↓: LP = UP; Visceral adipose tissue index↓: LP = UP; Total cholesterol↓: LP = UP HDL↑: LP < UP; LDL↓: LP = UP triglycerides↓: LP = UP; SBP↓: LP = UP DBP↓: LP = UP
		UP				Three different sessions were performed each week: Session 1 (3–5RM, 3′ rest); Session 2 (8–10RM, 1.5′ rest); Session 3 (20RM, 45″ rest), with daily undulation.	3	8	

5sCS, Five consecutive chair stand test; 6MWT, 6-min walk test; BM, body mass; BMI, body mass index; BW, body weight; CMJ, countermovement jump height; DBP, diastolic blood pressure; DUP, daily undulating periodization; F’TST, floor-to-stand test; GJST, Gallon jug shelf test; HDL, high-density lipoprotein; LP, linear periodization; LT, Laundry test; MVC, maximal voluntary contraction; NM, not mentioned; RM, repetition maximum; RT, resistance training; SBJ, standing broad jump; SBP, systolic blood pressure; TUG, timed up-and-go test, UP, undulating periodization; VJ, vertical jump; WUP, weekly undulating periodization.

### 3.3 Methodological quality of studies

Table 2 included an overview of the scores assessed using TESTEX. Median values regarding criteria matching were two (2–5) for study quality and seven (6–9) for study reporting. Overall, studies had a median score of 9 (8–12) out of the 15 possible points. Studies scored highly for eligibility criteria specified (*n* = 29); comparisons between-group at baseline and after invention (*n* = 29). In contrast, studies scored poorly in terms of specifying randomization procedure (*n* = 25), blinding of participants (*n* = 25) and assessors (*n* = 28). No authors reported adverse events in either training mode.

**Table 2:** Overview of the scores assessed by using TESTEX.

Study	Study quality criterion						Study reporting criterion								Overall
	1	2	3	4	5	Total	6	7	8	9	10	11	12	Total	
Baker et al. (1994) (38)	1	0	0	1	0	2	1	0	2	1	1	0	1	6	8
Rhea et al. (2002) (6)	1	0	0	1	0	2	1	0	2	1	1	0	1	6	8
Hoffman et al. (2003) (39)	1	0	0	1	0	2	2	0	2	1	1	0	1	7	9
Rhea et al. (2003) (40)	1	0	0	1	0	2	2	0	2	1	1	0	1	7	9
Buford et al. (2007) (41)	1	0	0	1	0	2	2	0	2	1	1	0	1	7	9
Peterson et al. (2008) (42)	1	0	0	1	0	2	2	0	2	1	1	0	1	7	9
Hartmann et al. (2009) (43)	1	0	0	1	0	2	2	0	2	1	1	0	1	8	10
Hoffman et al. (2009) (44)	1	0	0	1	0	2	2	0	2	1	1	0	1	7	9
Kok et al. (2009) (45)	1	1	1	1	0	4	2	0	2	1	1	1	1	8	12
Monteiro et al. (2009) (16)	1	0	0	1	0	2	2	0	2	1	1	1	1	8	10
Prestes et al. (2009) (7)	1	0	0	1	0	2	1	0	2	1	1	0	1	6	8
Vanni et al. (2009) (46)	1	0	0	1	0	2	1	0	2	1	1	0	1	6	8
Apel et al. (2011) (47)	1	0	0	1	0	2	1	0	2	1	1	0	1	6	8
Miranda et al. (2011) (48)	1	0	0	1	0	2	1	0	2	1	1	0	1	6	8
Lima et al. (2012) (49)	1	0	0	1	0	2	1	0	2	1	1	0	1	6	8
Simao et al. (2012) (50)	1	0	0	1	0	2	1	0	2	1	1	0	1	6	8
Ahmadizad et al. (2013) (13)	1	0	0	1	0	2	1	0	2	1	1	0	1	6	8
Franchini et al. (2015) (51)	1	0	0	1	0	2	1	0	2	1	1	0	1	6	8
Harries et al. (2015) (3)	1	1	0	1	0	3	2	1	2	1	1	1	1	9	12
Prestes et al. (2015) (52)	1	1	0	1	0	3	2	1	2	1	1	1	1	9	12
Ullrich et al. (2017) (53)	1	0	1	1	0	3	1	0	2	1	1	1	1	7	10
Buskard et al. (2018) (54)	1	1	1	1	1	5	1	0	2	1	1	1	1	7	12
Souza et al. (2018) (55)	1	0	1	1	0	3	1	0	2	1	1	1	1	7	10
Mahmoud et al. (2021) (56)	1	0	0	1	0	2	1	0	2	1	1	1	1	7	9
Rosell et al. (2021) (57)	1	0	0	1	0	2	1	0	2	1	1	1	1	7	9
Borges-Silva et al. (2022) (58)	1	0	0	1	0	2	1	0	2	1	1	1	1	7	9
Gonçalves et al. (2024) (59)	1	0	0	1	0	2	1	0	2	1	1	1	1	7	9
Lo’pez et al. (2024)	1	0	0	1	0	2	1	0	2	1	1	1	1	7	9
Riscart-López et al. (2024) (60)	1	0	0	1	0	2	1	0	2	1	1	1	1	7	9

Items for literature quality evaluation:1: Eligibility criteria specified (1 point); 2: Randomization specified (1 point); 3: Allocation concealment (1 point); 4: Groups similar at baseline (1 point); 5: Blinding of assessor (1 point); 6: Outcome measures assessed in 85% of patients (3 point); 7: Intention-to-treat analysis (1 point); 8: Between-group statistical comparisons reported (2 point); 9: Point measures and measures of variability for all reported outcome measures (1 point); 10: Activity monitoring in control groups (1 point); 11: Relative exercise intensity remained constant (1 point); 12: Exercise volume and energy expenditure (1 point).

### 3.4 Results of meta-analyses

#### 3.4.1 Athletic ability

##### 3.4.1.1 Upper and lower limb strength

**Figure 2:**
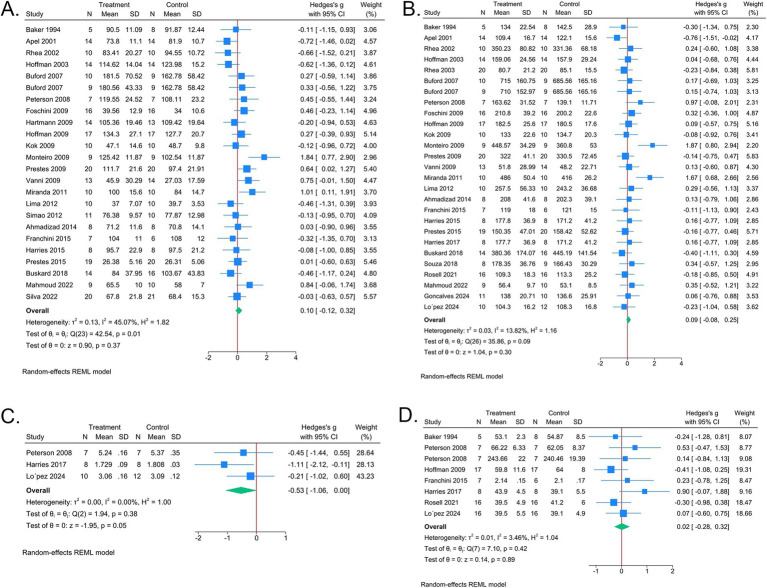
Forest plots of meta-analysis comparing athletic ability between LP and UP. **(A)** Upper limb push strength. **(B)** Lower limb squat strength. **(C)** Sprint speed. **(D)** Explosive power. Each square represents the estimate effect and 95% confidence interval (CI) of the study. The diamond represents the overall HR and 95% CI. The diamond on the left of *x* = 0 indicates that the effect of UP is lower than that of LP.

Twenty-three studies assessed the comparison between LP and UP on upper limb pushing strength. The results showed that LP and UP led to similar improvements in upper limb strength (SMD 0.08, 95% CI [−0.15, 0.31], Figure 2A). The *I*^2^ value was 46.12%, indicating moderate heterogeneity. The funnel plot was symmetrical (Supplementary Figure 1A, *p* of Egger’s test > 0.05), suggesting minimal risk of publication bias. Sensitivity analysis confirmed the robustness of the results (Supplementary Figure 2A). Subgroup analyses further revealed that the effect remained consistent across participants of different sexes, ages, training durations, and obesity levels (Table 3).

**Table 3:** Results of subgroup meta-analysis.

Items	Subgroups	*N* of study	Effect size (95%CI)	*I*^2^ (%)
Athletic ability				
*Upper limb push*	Overall	23	0.08 (−0.15, 0.31)	46.12
Sex	Man	13	0.11 (−0.25, 0.46)	56.62
	Woman	5	0.19 (−0.27, 0.66)	43.52
	Both	5	−0.07 (−0.48, 0.34)	31.34
Age	Adult	22	0.09 (−0.15, 0.33)	48.71
	Adolescent	1	−0.08 (−1.00, 0.85)	0
Duration	<8 weeks	—	—	—
	8–14 weeks	20	0.04 (−0.22, 0.30)	49.14
	>14 weeks	3	0.29 (−0.10, 0.68)	2.34
Training experience	Trained	12	0.03 (−0.34, 0.40)	58.32
	Not trained	11	0.13 (−0.15, 0.42)	30.97
Body mass status	Obesity	2	0.44 (−0.35, 1.23)	33.63
	Normally	21	0.05 (−0.19, 0.29)	47.24
*Lower limb squat*	Overall	25	0.08 (−0.10, 0.26)	20.31
Sex	Man	14	0.21 (−0.15, 0.57)	59.49
	Woman	5	0.07 (−0.27, 0.41)	0
	Both	6	−0.06 (−0.36, 0.32)	0
Age	Adult	23	0.08 (−0.12, 0.27)	28.2
	Adolescent	2	0.20 (−0.42, 0.83)	0
Duration	<8 weeks	—	—	—
	8–14 weeks	21	0.14 (−0.10, 0.37)	37.45
	>14 weeks	4	−0.06 (−0.39, 0.26)	0
Training experience	Trained	15	−0.02 (−0.22, 0.18)	0
	Not trained	10	0.25 (−0.09, 0.59)	39.90
Body mass status	Obesity	2	0.25 (−0.39, 0.88)	0
	Normally	23	0.07 (−0.12, 0.27)	20.31
*Explosive power*	Overall	7	−0.07 (−0.38, 0.24)	0
Sex	Man	6	0.02 (−0.32, 0.37)	0
	Woman	—	—	—
	Both	1	−0.41 (−1.08, 0.25)	0
Duration	<8 weeks		—	
	8–14 weeks	6	0.02 (−0.32, 0.37)	0
	>14 weeks	1	−0.41 (−1.08, 0.25)	—
Experience	Trained	5	−0.17 (−0.51, 0.18)	0
	Not trained	2	0.33 (−0.37, 1.03)	0
*Sprint speed*	Overall	2	−0.30 (−0.93, 0.33)	0
Training experience	Trained	1	−0.21 (−1.02, 0.60)	—
	Not trained	1	−0.45 (−1.44, 0.55)	—
Body composition				
*Body weight*	Overall	9	0.95 (−1.28, 3.17)	0
Sex	Man	5	0.07 (−2.76, 2.90)	0
	Woman	2	4.34 (−3.11, 11.79)	60.51
	Both	2	2.60 (−10.04, 15.24)	69.03
Age	Adult	8	1.13 (−1.12, 3.38)	0
	Adolescent	1	−7.00 (−21.79, 7.79)	0
Duration	<8 weeks	1	−2.80 (−9.44, 3.84)	—
	8–14 weeks	6	0.23 (−2.31,2.77)	0
	>14 weeks	2	8.99 (2.58, 15.40)	0
Body mass status	Obesity	2	0.26 (−2.84, 3.36)	0
	Normal	7	1.91 (−2.44, 6.25)	33.86
Training experience	Trained	5	−0.19 (−3.87, 3.50)	0
	Not trained	4	2.27 (−1.64, 6.18)	34.98
*BMI*	Overall	4	−0.12 (−1.35, 1.11)	48.26
Sex	Man	1	−0.20 (−1.92, 1.52)	—
	Woman	2	1.09 (−1.29, 3.48)	48.92
	Both	1	−1.30 (−2.56, −0.04)	0
Duration	<8 weeks	1	−1.30 (−2.56, −0.04)	—
	8–14 weeks	2	0.01 (−1.17, 1.19)	0
	>14 weeks	1	2.75 (−0.43, 5.93)	—
Body mass status	Obesity	2	0.01 (−1.17, 1.19)	0
	Normal	2	0.45 (−3.48, 4.38)	81.41
Training experience	Trained	1	−1.30 (−2.56, −0.04)	—
	Not trained	3	0.34 (−0.76, 145)	0
*Body fat percentage*	Overall	8	0.08 (−0.92, 1.07)	0
Sex	Man	3	0.32 (−0.98, 1.62)	0
	Woman	3	−0.05 (−2.39, 2.29)	44.29
	Both	2	−3.51 (−9.12, 2.09)	0
Age	Adult	7	0.20 (−0.82, 1.21)	0
	Adolescent	1	−3.10 (−8.35, 2.15)	0
Duration	<8 weeks	—	—	—
	8–14 weeks	7	−0.04 (−1.07, 0.98)	0
	>14 weeks	1	2.10 (−2.10, 6.30)	—
Body mass status	Obesity	1	0.60 (−1.52, 2.72)	—
	Normal	7	−0.16 (−1.42, 1.10)	9.40
Training experience	Trained	4	−1.06 (−3.74, 1.61)	26.79
	Not trained	4	0.27 (−1.05, 1.60)	1.18
*Fat-free body weight*	Overall	7	0.07 (−0.35, 0.49)	46.77
Sex	Man	4	−0.32 (−0.73, 0.09)	0
	Woman	3	0.51 (−0.02, 1.05)	29.6
Age	Adult	6	0.11 (−0.37, 0.59)	54.23
	Adolescent	1	−0.22 (−1.15, 0.71)	—
Duration	<8 weeks	—	—	—
	8–14 weeks	7	0.08 (−0.36, 0.52)	48.94
	>14 weeks	1	0.47 (−0.16, 1.09)	—
Body mass status	obesity	1	1.17 (0.23, 2.10)	0
	normal	6	−0.08 (−0.44, 0.29)	22.31
Training experience	trained	3	−0.21 (−0.76, 0.34)	0
	not trained	4	0.25 (−0.40, 0.91)	68.40
Blood lipid and blood glucose				
*Insulin resistance*	Overall	2	−1.36 (−4.56, 1.84)	93.38
Sex	Man	1	−3.04 (−4.44, −1.64)	—
	Woman	1	0.23 (−0.64, 1.09)	—
	Both	—	—	—
*Blood glucose*	Overall	3	0.00 (−0.43, 0.43)	0
Body mass status	Obesity	2	0.03 (−0.79, 0.85)	0
	Normally	1	−0.01 (−0.51, 0.49)	—

Twenty-five studies evaluated the comparison between LP and UP on lower limb squat strength. No statistically significant difference was observed between LP and UP (SMD 0.08, 95% CI [−0.10, 0.26], Figure 2B). The *I*^2^ value was 20.31%, indicating low heterogeneity. The funnel plot was asymmetric (*p* of Egger’s test <0.05, Supplementary Table 1), suggesting potential publication bias risk. Sensitivity analysis showed the result was robust (Supplementary Figure 2B). Subgroup analyses also found no significant differences across participants of different sexes, ages, or training durations (Table 3).

##### 3.4.1.2 Sprint speed and explosive power

There were two and seven studies designed comparison between LP and UP on sprint speed and explosive power, respectively. The results showed that no significant difference between LP and UP was found on sprint speed (SMD −0.30, 95% CI [−0.93, 0.33], Figure 2C) and explosive power (SMD 0.07, 95% CI [−0.35, 0.49], Figure 2D). Funnel plot and sensitivity analysis showed the result was robust (Supplementary Figures 1, 2).

#### 3.4.2 Body composition

**Figure 3:**
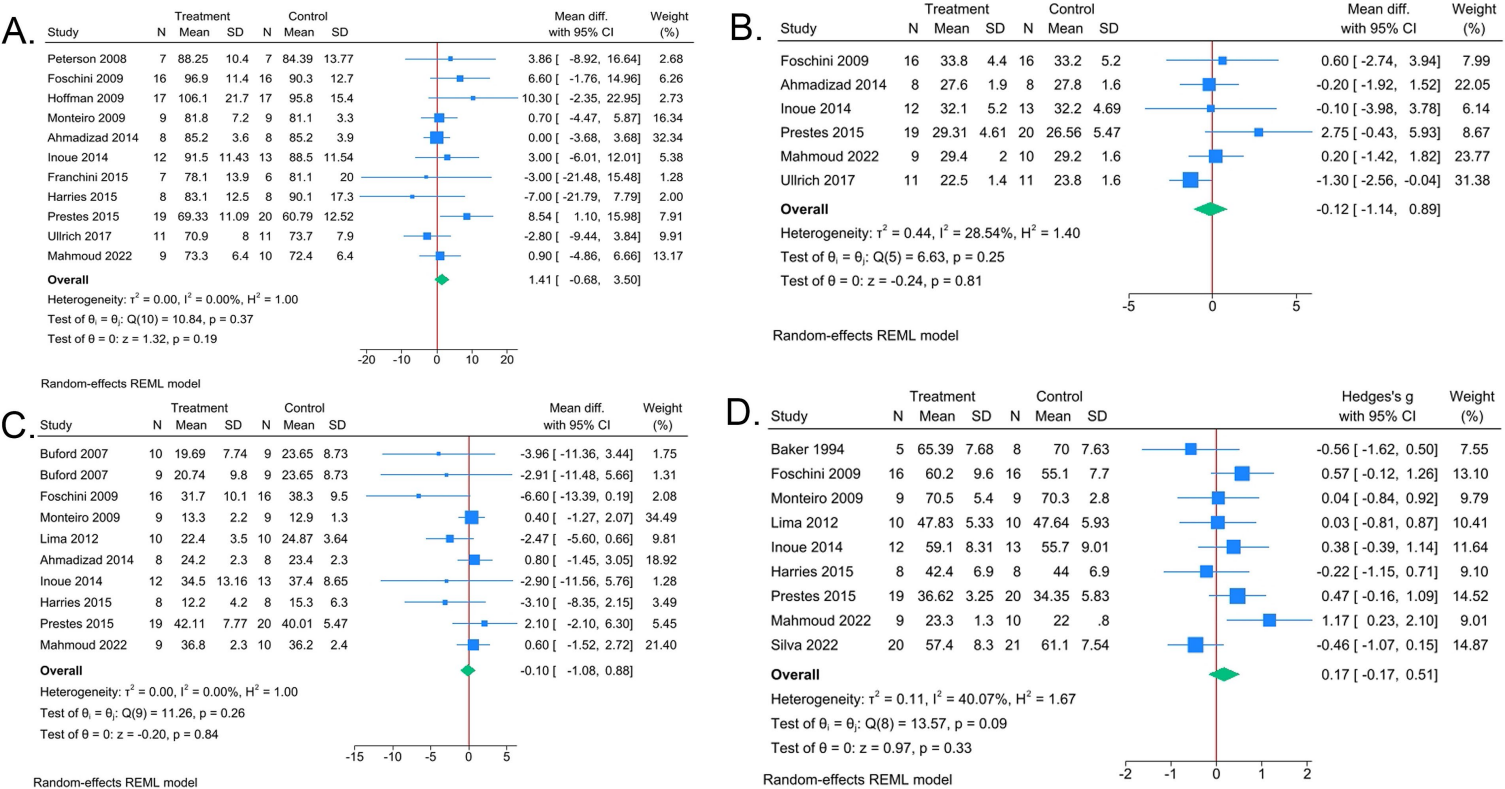
Forest plots of meta-analysis comparing body composition between LP and UP. **(A)**. Body weight. **(B)** BMI. **(C)** Body fat percentage. **(D)** Fat-free body weight. Each square represents the estimate effect and 95% confidence interval (CI) of the study. The diamond represents the overall HR and 95% CI. The diamond on the left of *x* = 0 indicates that the effect of UP is lower than that of LP.

Nine, four, eight, and seven studies reported body weight, BMI, body fat percentage, and fat-free body weight, respectively. Overall, LP and UP had a similar effect on body composition, including body weight, BMI, body fat percentage, and fat-free body weight (Figure 3). Funnel plots indicated little bias on for body weight, body fat percentage, and fat-free body weight (*p* of Egger’s test > 0.05, Supplementary Figure 3), but a potential publication bias was detected when assessed BMI (*p* of Egger’s test <0.05, Supplementary Table 1). Sensitivity analyses showed the results were robust (Supplementary Figure 4). Further subgroup analyses found that when the training duration exceeded 14 weeks, LP demonstrated a more pronounced effect on weight loss (MD 8.99, 95%CI [2.58–15.40], Table 3), however, when training duration was less than 8 weeks, LP reduced more than UP on BMI (MD −1.30, 95% CI [−2.56 to −0.04], Table 3). And UP was more effective on increasing fat-free body weight in obese participants (SMD 1.17, 95% CI [0.23–2.10], Table 3).

#### 3.4.3 Blood lipid and blood glucose

##### 3.4.3.1 Blood lipid and blood pressure

Only one study compared blood pressure and blood lipid, including triglyceride, total cholesterol, LDL, and high-density lipoprotein (HDL) from old participants (aged 64 ± 2.1 years) (15). The research findings demonstrated that, compared to LP, UP exhibited a more evident effect in ameliorating triglycerides, total cholesterol, LDL, and HDL in the older adults, particularly in reducing blood pressure.

##### 3.4.3.2 Blood glucose and insulin resistance

**Figure 4:**
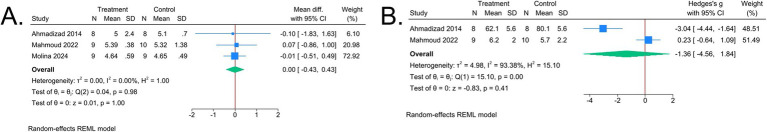
Forest plots of meta-analysis comparing blood lipid and blood glucose between LP and UP. **(A)** Blood glucose. **(B)** Insulin resistance. Each square represents the estimate effect and 95% confidence interval (CI) of the study. The diamond represents the overall HR and 95% CI. The diamond on the left of *x* = 0 indicates that the effect of UP is lower than that of LP.

Three and two studies evaluated blood glucose and insulin resistance indicators, respectively. Overall, there was no statistically significant difference between LP and UP on blood glucose (MD 0.00, 95% CI [−0.43 to 0.43], Figure 4A) and insulin resistance (MD −1.36, 95% CI [−4.56, 1.84], Figure 4B). Sensitivity analyses confirmed the stability of the overall results (*p* of Egger’s test > 0.05, Supplementary Figure 5 and Supplementary Table 1). Subgroup analysis revealed that UP exhibited a more pronounced effect in reducing insulin resistance among men, (SMD −3.04, 95% CI [−4.44, −1.64], Table 3).

## 4 Discussion

The findings of this meta-analysis revealed that LP and UP had similar effects on improving athletic capacity, improving body composition, and regulating blood glucose and insulin resistance. Further subgroup results indicated that UP is superior to LP in increasing lean body mass in obese individuals and setting short-term weight loss goals, but LP is more suitable for long-term weight loss. The only male study included indicated that UP was superior to LP in reducing insulin resistance in males.

This study assessed the effects of two load-application methods on maximum upper- and lower-limb strength via pushing and squatting exercises, respectively. Our findings revealed no significant difference in augmenting upper- and lower-limb maximal strength, aligning with the results of previous meta-analyses (3, 10). Conversely, Caldas et al. (9) reported that UP was superior for increasing maximal strength, though not for power and muscle endurance. They hypothesized that the disparity in the number of included studies contributed to the variation in effect size. An alternative explanation posited by subsequent investigators concerns the analytical approach: calculating SMD based solely on post-intervention values without accounting for within-group pre-post changes may have introduced methodological bias (10). Previous research has pointed out that relatively small sample sizes may impose limitations. Building on prior studies, this research incorporated a larger sample, further validating the robustness of the comparable strength-improvement effects between UP and LP. In the studies by Harris et al. (3) and Caldas et al. (9) most of the included studies had short training durations. Some research posited that UP could significantly enhance muscle strength in the early training stages, while LP was more effective in the later stages (6, 16). The present study addressed this limitation by incorporating a greater proportion of long-term training investigations and conducting stratified subgroup analyses by intervention duration. Our results demonstrated similar strength-enhancing effects of LP and UP across varying training periods, suggesting that muscular strength continues to accrue with training progression regardless of whether loads are arranged linearly or in undulating fashion. Currently, mechanistic investigations into muscular strength adaptations elicited by distinct RT periodization strategies remain limited. Previous research indicates that periodization models do not differentially influence fundamental adaptive mechanisms underlying resistance training responses (17, 18). Accordingly, we posit that both LP and UP stimulate muscular development through progressive overload, thereby engendering comparable neuromuscular and morphological adaptations, notwithstanding their divergent load distribution patterns. To reveal the effects of two different training load regiments on different people, this study also conducted subgroup analyses based on participants’ age and sex. Results indicated consistent training effects between LP and UP among both adolescents and adults, as well as between male and female participants. Notably, Moesgaard et al. (10) identified training status as a critical moderator, demonstrating that UP enables greater customization of training loads according to individual capabilities, thereby facilitating superior strength gains in trained individuals. Conversely, untrained participants demonstrate significant adaptive responses even to lower intensities (10–12 repetition maximum). Diverging from Moesgaard et al.’s (10) findings, we observed similar strength improvements between LP and UP regardless of training experience. This discrepancy may be attributable to the relatively extended intervention durations characterizing studies in our analysis. From a long-term adaptation perspective, LP and UP appear to elicit comparable muscular adaptations irrespective of participant training status. It is noteworthy that despite subgroup stratification by training level, residual heterogeneity persisted. Therefore, we contend that load arrangement pattern does not constitute the primary determinant of strength development; rather, individuals with varying training backgrounds may respond differentially to specific loading parameters. Further classification based on competitive level warrants investigation. The application of strength training in enhancing speed and explosive power has been widely adopted, yet there is still relatively little attention paid to the load arrangement mode of periodic training. Our results show that the effects of LP and UP in terms of speed and explosive power are similar with little heterogeneity, indicating that there is little difference between the two load regimens in terms of their effects on improving speed and explosive power. Changes in maximal squat strength reflected in improvements in short sprint performance (19), no difference between two load regimens on lower limb squat strength could be the reason that it was similar on speed. When conducting RT combined speed training, using DUP to arrange RT has a slight advantage over LP in terms of speed improvement (20).

The goals of improving body composition are decreasing overlarge body fat and increasing lean body mass, especially for obese population. The advantage of periodic RT lies in the fact that, through a relatively long period of RT, it stimulates muscle growth, reduces visceral fat, and further increases the resting metabolic rate, thereby more effectively improving body composition (21). Overall, there was no difference in the overall change effects of LP and UP in terms of body mass, body fat and lean body mass. Further subgroup analysis revealed that when the training period was short (less than 8 weeks), UP was superior to LP in weight reduction, while when the training period was longer (longer than 14 weeks), LP was better at regulating weight than UP. LP typically employs progressive unidirectional load manipulation, with volume and intensity modulated sequentially in fixed trajectories—generally prioritizing volume accumulation before intensity progression. Conversely, UP incorporates multiple concurrent training objectives, simultaneously stimulating adaptations in hypertrophy, strength, and power through flexible, concurrent adjustments of both volume and intensity parameters. Based on these differential temporal responses, we posit that underlying mechanisms involve distinct responsiveness of bioactive signaling cascades to load intensity configurations (22). Specifically, UP appears to confer superior short-term improvements in body composition, potentially attributable to its capacity for rapid and flexible modulation of training stimuli within condensed timeframes. Yet, as training progresses, LP shifts from high-volume/low-intensity to low-volume/high-intensity, optimizing adaptation. Beyond hypertrophy and strength, LP’s initial high volume more effectively expands aerobic capacity (23), mitochondrial content (24, 25), and fatigue resistance (26). All of these may further strengthen the function of fat oxidation, thereby demonstrating a better weight control effect compared to UP. For trained participants, our results found that UP was superior than LP on body mass regulation, though this observation derived from a single study. We speculated that more frequent variation of training volume and intensity was needed and beneficial to drive neurophysiological adaptations in trained individuals by assigning UP scheme. However, for untrained individuals, any type of periodization arrangement was sufficient to generate adequate training stimulation, and there was little need to frequently change training volume and intensity. It is worth noting that one study included indicated that obese participants could benefit more on increasing lean body mass by UP than LP. Obese individuals may cause metabolic disorders in skeletal muscles, manifested as oxidative stress and increased glycated hemoglobin levels, insulin resistance and other aspects (27–29). Through RT, the function of skeletal muscles can be improved, thereby reversing the multiple pathological changes caused by obesity (30, 31). However, for participants with normal weight, the corresponding changes may be blunted. The improvement of indicators is also related to the load intensity of RT. The effect of higher-intensity RT is superior to lower-intensity RT (31), and that’s the potential reason that the effect of UP is better than LP. Collectively, the mechanistic pathways through which RT periodization influences body composition remain incompletely elucidated. RT likely mediates its effects through diverse bioactive signaling molecules, warranting further mechanistic investigation in future research.

Prior research targeting hyperglycemia, dyslipidemia, and hypertension has predominantly emphasized aerobic exercise interventions. However, RT independently confers beneficial metabolic effects, and combined aerobic and resistance training yields synergistic improvements (32–34). For people with limited mobility, RT is easier to implement than aerobic exercise (35). Nevertheless, optimal load arrangement strategies for RT-mediated improvements in glycemic control, blood pressure regulation, and lipid profiles remain inadequately characterized. Previous investigations suggest that low-to-moderate intensity RT may confer superior benefits for lipid profile modification compared to high-intensity protocols (36). Moreover, when equalizing the training load by decreasing the number of sets and repetitions to offset the increased weight being lifted, there is limited additional advantage in increasing the RT intensity (37). A more substantial reduction in acute blood glucose levels was observed in high intensity RT compared to moderate intensity. The present findings demonstrated no significant differences between LP and UP in effects on blood lipids, glycemic parameters, or blood pressure. These results indicate that both periodization strategies effectively improve cardiometabolic risk factors when implemented as structured training programs. The fundamental mechanisms through which RT improves blood lipid profiles, glucose homeostasis, and blood pressure regulation appear to center on increments in lean muscle mass and enhanced muscular capacity for lipid and glucose metabolism regulation. Given that LP and UP elicit comparable hypertrophic and functional adaptations, these parallel neuromuscular outcomes likely account for their similar metabolic effects. Subgroup analysis revealed that UP was superior to LP in improving insulin resistance among men, yet only one study included older participants. Future research is required to confirm the potential sex-related differences associated with various training load regimens.

In the present meta-analysis, the majority of outcome measures exhibited low statistical heterogeneity; however, insulin resistance demonstrated substantial heterogeneity. We identified three potential sources of this heterogeneity. First, considerable variability existed in training protocols across studies, with intervention durations ranging from 6 to 28 weeks and training frequencies from 2 to 5 sessions per week, which may have contributed to between-study variance. Second, participant characteristics differed markedly regarding sex, age, training experience, and adiposity status—factors known to influence training responsiveness. Third, heterogeneity may have arisen from methodological diversity in outcome assessment, including variations in measurement techniques and reporting units. Notably, for outcomes assessed in a limited number of primary studies (e.g., insulin resistance), high heterogeneity estimates are particularly susceptible to bias and instability. Consequently, additional well-powered investigations are warranted to generate more robust and generalizable findings for these parameters.

Study quality and reporting completeness were evaluated using the TESTEX checklist. Overall, the methodological quality of included articles ranged from moderate to high. Notable deficiencies were observed regarding the specification of randomization procedures and the blinding of participants and outcome assessors. Although several studies reported the use of random allocation, the specific randomization methodology was not detailed. Given that adequate randomization constitutes a critical safeguard for internal validity in controlled trials, future investigations should strictly adhere to and explicitly report these procedures to enhance methodological rigor. Regarding allocation concealment, the inherent nature of exercise training interventions—where participants actively engage in prescribed protocols—renders this criterion particularly challenging to fulfill, accounting for the relatively low scores in this domain. With respect to assessor blinding, particular attention should be devoted to implementation and transparent reporting in future investigations to strengthen the quality of randomized controlled trials. Additionally, incomplete reporting of adverse events and imprecise documentation of training intensity control measures were observed. Comprehensive detailing of methodological controls and secondary outcomes in future reports would facilitate more transparent and reproducible research presentation.

This study has several strengths. First, compared with previous meta-analyses focused on maximal strength, the present investigation incorporated a substantially greater number of long-term intervention studies. Second, the outcome measures were not restricted to strength parameters; rather, a comprehensive array of health-related indicators was included. To our knowledge, this represents the first meta-analysis comparing the effects of UP and LP on blood pressure reduction, glycemic control, and lipid profile improvement. These findings provide an empirical foundation for developing evidence-based exercise prescriptions targeting cardiovascular and metabolic health. Third, comprehensive subgroup analyses were conducted with stratification by intervention duration, training experience, and adiposity status, thereby identifying optimal load configurations for distinct training objectives. Fourth, rigorous methodological standards were maintained through adherence to PRISMA guidelines and application of the TESTEX tool for quality appraisal and risk of bias assessment. Fifth, detailed, clinically relevant practical recommendations were formulated based on the synthesized evidence, offering actionable guidance for individuals with diverse resistance training requirements and health optimization goals.

This study also has several limitations. First, the training status of included participants exhibited substantial heterogeneity, and the granularity of training-level categorization was insufficient. This may have introduced unmeasured variability and potential bias. Second, the number of eligible primary studies was relatively limited for certain outcomes, potentially compromising the robustness of pooled estimates; consequently, subgroup findings require confirmation through additional investigations. Third, some subgroup analyses were based on scant evidence, with certain stratifications comprising only one or two primary studies (e.g., fat-free mass in obese individuals; sex-specific differences in insulin resistance). These results should be interpreted with considerable caution, as they may be unstable and can offer only preliminary, hypothesis-generating insights. Future research with expanded sample sizes and more refined stratification criteria is warranted to substantiate these preliminary observations.

This study offers practical applications for designing the working load of RT to achieve various goals among participants with different characteristics. When the objective is to enhance competitive ability, both UP and LP are reasonable options for load arrangement. For example, a 12-week program for team-sport athletes could effectively use either an LP model or a UP model to improve strength and power. In terms of improving competitive-related factors such as upper and lower limb maximal strength, sprinting ability, and explosive power, UP and LP play equivalent roles. In fact, these two methods can be combined when formulating the load scheme. A practical approach is to employ an LP block for foundational strength over 8–12 weeks, followed by a UP block to concurrently develop multiple athletic qualities in the pre-competition phase. Overall, there is no significant difference between UP and LP in improving body composition. For shorter-duration training (e.g., 6–8 week interventions), UP yields more obvious effects on weight/BMI reduction. For longer-duration training (e.g., >14 weeks), LP is recommended for sustained weight management. Regarding obese participants, UP is more conducive to increasing lean body mass. Thus, for an obese individual seeking body recomposition, an 8-week UP program (alternating intensity zones across sessions) is preferable. For experienced trainees, UP may provide better results in body mass regulation due to its varied stimulus. An advanced lifter on a plateau might switch to UP to re-stimulate adaptation. RT can improve blood pressure, blood lipids, and blood glucose, and UP and LP have equivalent effects in these aspects. Therefore, for general health improvement in metabolic syndrome, either model can be chosen based on client preference. For men aged over 65 years, the UP approach may have certain advantages in enhancing insulin sensitivity. A feasible application is a whole-body DUP program (e.g., alternating strength, hypertrophy, and endurance sessions) for men aged over 65 years with insulin resistance.

## 5 Conclusion

LP and UP had similar effects on improving athletic capacity, improving body composition, and regulating blood glucose and insulin resistance. UP is superior to LP in increasing lean body mass in obese individuals and setting short-term weight loss goals, but LP is more suitable for long-term weight loss.

## Data Availability

The original contributions presented in the study are included in the article/Supplementary material, further inquiries can be directed to the corresponding authors.
