# Supplementary material for: Comparison of linear and undulating periodization resistance training on athletic capacities and health promotion: a systematic review and meta-analysis
Source: Front Public Health. 2026 Mar 5;14:1707627. doi: 10.3389/fpubh.2026.1707627 (PMC12999919; doi:10.3389/fpubh.2026.1707627)
Supplement: Supplementary file 1 [file Supplementary_file_1.docx]

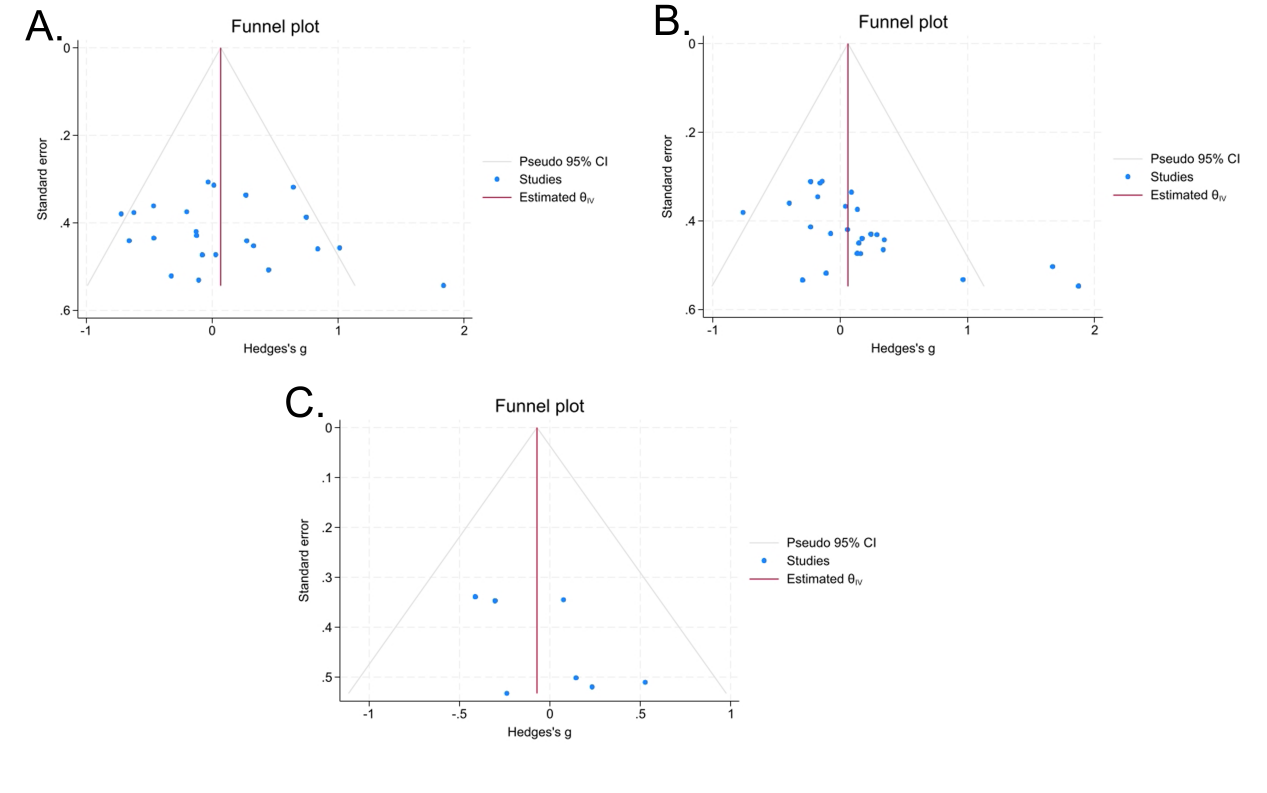


Supplemental Figure 1. Funnel plot of meta-analysis comparing athletic ability between LP and UP. A. upper limb push strength. B. lower limb squat strength. C. Explosive power. Funnel plot for athletic ability including 95% CI lines. The vertical line represents zero size. SMD represents the standard mean difference.


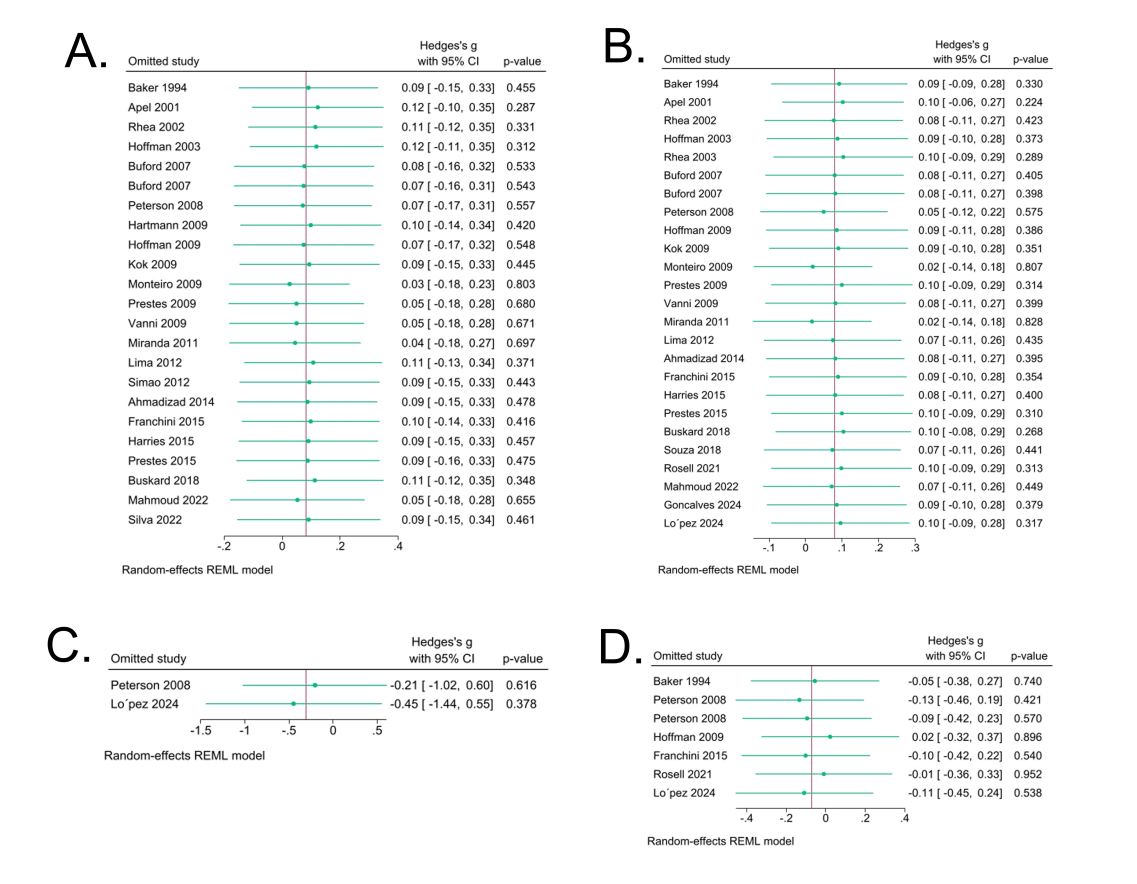


Supplemental Figure 2. Sensitivity analysis of meta-analysis comparing athletic ability between LP and UP. A. upper limb push strength. B. lower limb squat strength. C. Explosive power. Each circle represents the estimated effect and 95% confidence interval (CI) when the study was excluded.


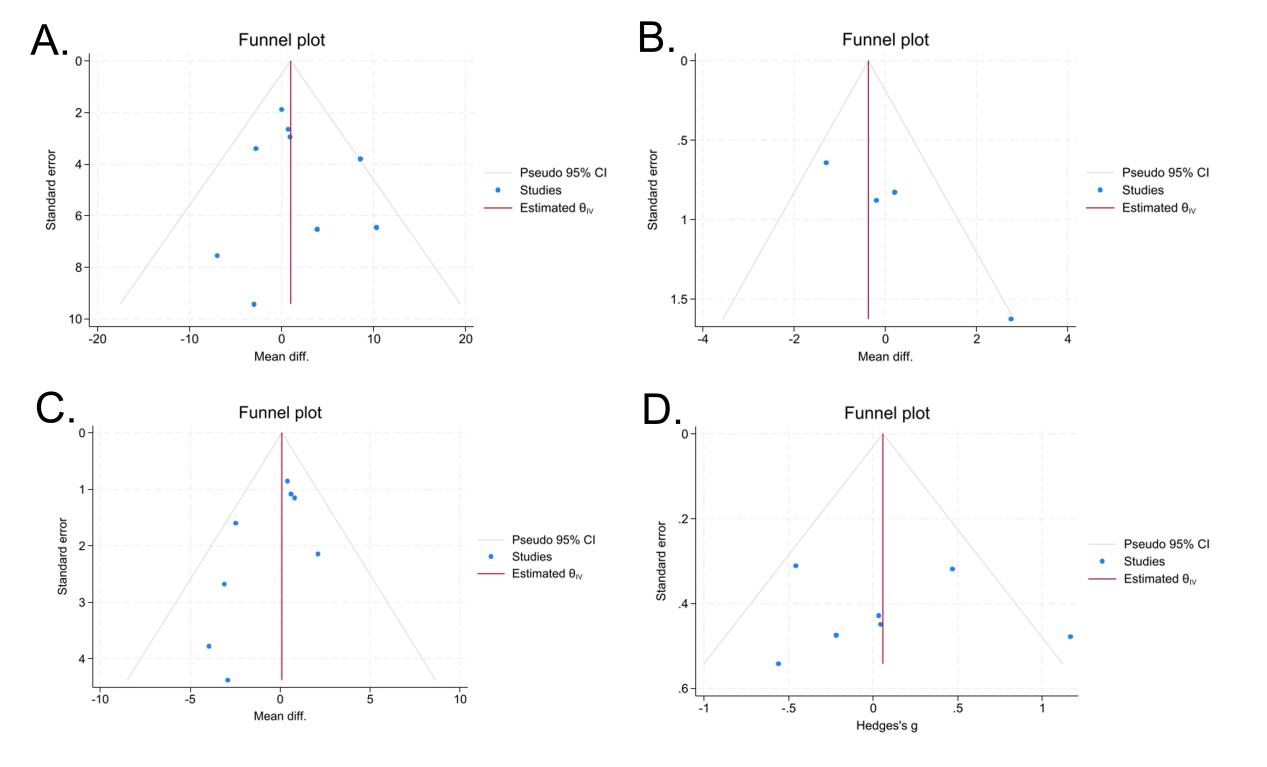


Supplemental Figure 3. Funnel plot of meta-analysis comparing body composition between LP and UP. A. Body weight. B. BMI. C. Body fat percentage. D. Fat-free body weight. Funnel plot for body composition including 95% CI lines. The vertical line represents zero size. SMD represents the standard mean difference and MD represents mean difference.


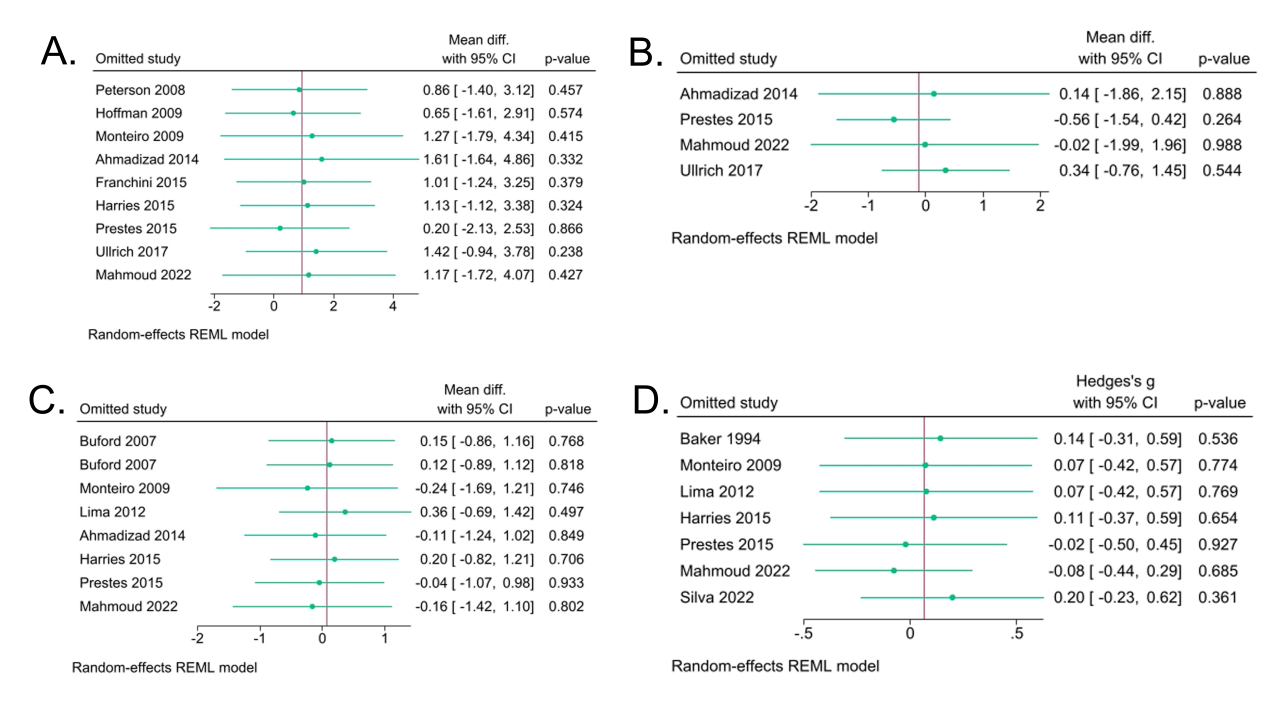


Supplemental Figure 4. Sensitivity analysis of meta-analysis comparing body composition between LP and UP. A. Body weight. B. BMI. C. Body fat percentage. D. Fat-free body weight. Each circle represents the estimated effect and 95% confidence interval (CI) when the study was excluded.


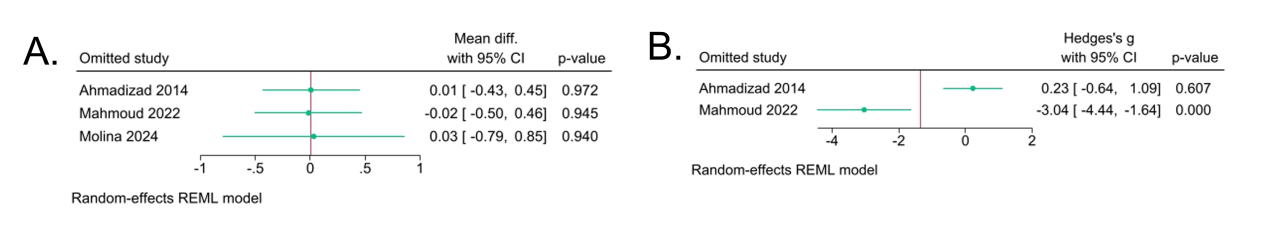


Supplemental Figure 5. Sensitivity analysis of meta-analysis comparing blood lipid and blood glucose between LP and UP. A. Blood glucose. B. Insulin resistance. Each circle represents the estimated effect and 95% confidence interval (CI) when the study was excluded.
