# Supplementary material for: Comparison of linear and undulating periodization resistance training on athletic capacities and health promotion: a systematic review and meta-analysis
Source: Front Public Health. 2026 Mar 5;14:1707627. doi: 10.3389/fpubh.2026.1707627 (PMC12999919; doi:10.3389/fpubh.2026.1707627)
Supplement: Supplementary file 2 [file Table_1.docx]

| **Outcomes** | **P value** |
| --- | --- |
| **Athletic ability** |  |
| *Upper limb push* | 0.411 |
| *Lower limb squat* | 0.002 |
| *Explosive power* | 0.149 |
| *Sprint speed* | - |
| **Body composition** |  |
| *Body weight* | 0.657 |
| *BMI* | 0.043 |
| *Body fat percentage* | 0.074 |
| *Fat-free body weight* | 0.879 |
| **Blood lipid and blood glucose** |  |
| *Insulin resistance* | - |
| *Blood sugar* | 0.935 |

Supplemental Table 1. Results of Egger's test statistics for each outcome.
